# Quantifying quantum coherence with quantum Fisher information

**DOI:** 10.1038/s41598-017-15323-7

**Published:** 2017-11-14

**Authors:** X. N. Feng, L. F. Wei

**Affiliations:** 1 State Key Laboratory of Optoelectronic Materials and Technologies, School of Physics, Sun Yat-Sen University, Guangzhou, 510275 China; 2 Information Quantum Technology Laboratory, School of Information Science and Technology, Southwest Jiaotong University, Chengdu, 610031 China

## Abstract

Quantum coherence is one of the old but always important concepts in quantum mechanics, and now it has been regarded as a necessary resource for quantum information processing and quantum metrology. However, the question of how to quantify the quantum coherence has just been paid the attention recently (see, e.g., Baumgratz *et al*. PRL, 113. 140401 (2014)). In this paper we verify that the well-known quantum Fisher information (QFI) can be utilized to quantify the quantum coherence, as it satisfies the monotonicity under the typical incoherent operations and the convexity under the mixing of the quantum states. Differing from most of the pure axiomatic methods, quantifying quantum coherence by QFI could be experimentally testable, as the bound of the QFI is practically measurable. The validity of our proposal is specifically demonstrated with the typical phase-damping and depolarizing evolution processes of a generic single-qubit state, and also by comparing it with the other quantifying methods proposed previously.

## Introduction

Originally, the concept of coherence was introduced to describe the interference phenomenon among waves. In recent years, quantum coherence has been paid much attention, as it is a necessary resource for various quantum engineerings, e.g., quantum key distributions^1^, quantum computation^2^, and quantum metrology^3^, etc. Indeed, the basic advantage of the quantum information processing over the classical counterpart is based on the utilizations of quantum coherence.

Quantum coherence is a fundamental phenomenon in quantum physics. However, as one of the important physical resources, its measurement is not easy to be defined. In fact, in recent years various functions such as the fidelity based distance measurement^4^, trace distance^5^, relative entropy^6^, quantum correlation^7,8^, and the skew information^9,10^ etc., have been suggested to quantify the quantum coherence. With these measurements, certain properties of quantum coherence, typically, e.g., the distillation of coherence^11,12^ and the nonclassical correlations^7,8,13^, have been described. Note that the quantum correlation has been well measured by quantum discord^14^ and other distance functions^15,16^. Furthermore, quantum entanglement, as a specifical representation of the quantum correlation in various multipartite quantum systems, has been quantified both pure axiomatically and experimentally^17–20^. The former is achieved by introducing some mathematical functions, such as the entanglement entropy^17^, entanglement of distillation^12^, and entanglement cost^19^, etc. While, the latter one was implemented by measuring the violations of the Bell-type inequalities, although certain exceptional cases wherein the non-locality vanishes but entanglement persists, still exist^21^.

A basic question is, how to generically quantify the quantum coherence carried by an arbitrary quantum state of a quantum system^4,6,9,22^? Interestingly, Baumgratz *et al* ^6^. pointed out that, any quantity $C(\rho )$ for effectively measuring the amount of quantum coherence in a quantum state $\rho$ should satisfy the following conditions:

(C1) It should be non-negative and vanishes if and only if the state is incoherent, i.e. $C(\rho )\ge 0$ and $C(\rho )=0$ iff $\rho \in$ $\Pi$ with $\Pi$ being the set of incoherent states.

(C2a) It should be non-increasing under any incoherent completely positive and trace preserving (ICPTP) operation, i.e., $C(\rho )\ge {C}_{ICPTP}(\rho )$; or (C2b) More strictly, it should be monotonic for average under subselection based on the measurement outcomes, i.e., $C(\rho )\ge \sum {p}_{n}C({A}_{n}\rho {A}_{n}^{\dagger }/{p}_{n})$; and

(C3) It should be convex, i.e., contractive under mixing of quantum states; $\sum {p}_{n}C({\rho }_{n})\ge C(\sum {p}_{n}{\rho }_{n})$ for any ensemble $\{{p}_{n},{\rho }_{n}\}$.

In what follows, these conditions will be called as Baumgratz *et al*.’s criticism for simplicity. It is easy to verify that, once the conditions (C2b) and (C3) are satisfied simultaneously, the condition (C2a) is satisfied naturally. Above, the ICPTP operation maps an incoherent state to another incoherent state. It is defined as follows. Generally, any quantum operation ${\rm{\Phi }}(\rho )$ performed on the quantum state $\rho$ can be written as

1 $${\rm{\Phi }}(\rho )=\sum _{\mu }{A}_{\mu }\rho {A}_{\mu }^{\dagger },$$

in the Kraus representation, wherein $\{{A}_{\mu }:\,\,{\sum }_{\mu }{A}_{\mu }^{\dagger }{A}_{\mu }=I\}$ are the complete-positive-trace-preserving operators. Also, any incoherent state can always be expressed as $\delta ={\sum }_{i\mathrm{=1}}^{d}{p}_{i}|i\rangle \langle i|$, with the zero off-diagonal elements. Furthermore, if the operation ${A}_{\mu }$ satisfies the condition^6^: ${A}_{\mu }\hat{\delta }{A}_{\mu }^{\dagger }\in$ ${\rm{\Pi }}$, with ${\rm{\Pi }}$ denoting the set of incoherent states for an arbitrary $\hat{\delta }\in$ ${\rm{\Pi }}$ and $\mu$, then ${A}_{\mu }$ is an ICPTP and reads^23^: ${A}_{\mu }={\sum }_{k,l}^{n}{a}_{kl}|k\rangle \langle l|$, wherein every $k\le n$ occurs at most once. Obviously, the operator ${A}_{\mu }$ maps a diagonal matrix to another diagonal one. In this sense, the usual dephasing, depolarizing, phase-damping and amplitude-damping processes can be treated as the incoherent operations, respectively.

Besides various measurements proposed previously, in this paper we introduce another quantity, i.e., the quantum Fisher information (QFI), to generically quantify the quantum coherence. As every ICPTP operation can be obtained from a partial trace on an extended system under certain unitary transformations^24^, we specifically show that, the QFI satisfies the Baumgratz *et al*.’s criticism. Since QFI is also mathematically related to some other functions proposed previously, such as the relative entropy^25^, fidelity based on the distance measurement^26^, and the skew information^27^ etc., for quantifying the quantum coherence, it is logically reasonable^28^ by using the QFI to quantify the quantum coherence. However, differing from most of the pure axiomatic functions proposed previously, the present proposal by using the QFI to quantify quantum coherence is experimentally testable, as the lower- and upper bounds of the QFI are practically measurable. The validity of our proposal will be demonstrated specifically with the evolutions of a generic one-qubit state under the typical phase-damping and depolarizing processes, respectively.

## Quantum Fisher information and its Properties

For completeness, we briefly review QFI and some of its properties^29^, which will be utilized below to prove our arguments.

As we know that some of physical quantities are not directly accessible but can only be indirectly estimated from the measurement outcomes of the other observable(s). The quantum estimation theory has been developed to focus the relevant parameter estimation problems. Typically, the well-known Cramér-Rao inequality^26^ states that the lower bound of the variance of the estimated quantity $\theta$ should be limited by

2 $${({\rm{\Delta }}{\theta })}^{2}\ge \frac{1}{{F}_{{Q}}({\rho }_{{\theta }})},$$

with ${F}_{Q}({\rho }_{\theta })$ being the QFI of the quantum state ${\rho }_{\theta }$. Therefore, the QFI plays a very important role in quantum metrology and determines the reachable accuracy of the estimated quantity. Historically, there are several definitions of the QFI from different perspectives, see, e.g.,^27^. In quantum metrology, in term of the selfadjoint operator symmetric logarithmic derivative (SLD) ${L}_{\theta }$ ^29^, defined by

3 $$\frac{{\rho }_{\theta }{L}_{\theta }+{L}_{\theta }{\rho }_{\theta }}{2}=\frac{\partial {\rho }_{\theta }}{\partial \theta },$$

for a quantum state ${\rho }_{\theta }$ with a parameter $\theta$ being estimated, the QFI is generically defined as

4 $${F}_{Q}({\rho }_{\theta })=Tr[{\rho }_{\theta }{L}_{\theta }^{2}\mathrm{].}$$

Note that the equation (3) is a Lyapunov matrix equation, whose generic solution can be written as

5 $${L}_{\theta }=2{\int }_{0}^{\infty }dt\exp \{-{\rho }_{\theta }t\}{\partial }_{\theta }{\rho }_{\theta }\exp \{-{\rho }_{\theta }t\mathrm{\}.}$$

By writing ${\rho }_{\theta }$ in its eigenbasis, i.e., ${\rho }_{\theta }={\sum }_{i}{\mu }_{i}|{\mu }_{i}\rangle \langle {\mu }_{i}|$, such a generic solution can be specifically expressed as

6 $${L}_{\theta }=2\sum _{ij}\frac{\langle {\mu }_{j}|{\partial }_{\theta }{\rho }_{\theta }|{\mu }_{i}\rangle }{{\mu }_{i}+{\mu }_{j}}|{\mu }_{j}\rangle \langle {\mu }_{i}\mathrm{|.}$$

As a consequence, with Eq. (4) the QFI is given by

7 $${F}_{Q}(\theta )\,=\,2\sum _{ij}\frac{|\langle {\mu }_{j}|{\partial }_{\theta }{\rho }_{\theta }|{\mu }_{i}\rangle {|}^{2}}{{\mu }_{i}+{\mu }_{j}}\mathrm{.}$$

Physically^30,31^, the parameter $\theta$ expected to be estimated coincides with a global phase. It can be encoded by applying a unitary transformation: ${U}_{\theta }=\exp (-i\theta H)$, to a quantum state, i.e.,

8 $$\rho \to {\rho }_{\theta }={U}_{\theta }\rho {U}_{\theta }^{\dagger }\mathrm{.}$$

Here, $H$ is the Hamiltonian of the quantum system with the initial state $\rho$. Typically, $H$ is assumed to be independent from $\theta$. As a consequence, Eq. (7) becomes^26^:

9 $${F}_{Q}(\rho ,H)=2\sum _{i,j}\frac{{({\lambda }_{i}-{\lambda }_{j})}^{2}}{{\lambda }_{i}+{\lambda }_{j}}|{H}_{ij}{|}^{2},\,\,{H}_{ij}=\langle {\lambda }_{i}|H|{\lambda }_{j}\rangle $$

with $\{{\lambda }_{i},|{\lambda }_{i}\rangle \}$ being the eigenvalues and the corresponding eigenvectors of the density operator $\rho$, respectively.

It is proven that the QFI possesses some important properties. First, it is additive under tensoring^32^, i.e.,

10 $${F}_{{Q}}({\rho }_{A}\otimes {\rho }_{{B}},{H}_{{A}}\otimes {{I}}_{{B}}+{I}_{A}\otimes {H}_{B})={F}_{Q}({\rho }_{A},{H}_{A})+{F}_{Q}({\rho }_{B},{H}_{B}),$$

for a composite system $A+B$. Next, it is unchanged under any unitary transformation $U$ commuting with the Hamiltonian $H$ ^32^, i.e.,

11 $${F}_{Q}(\rho ,H)={F}_{Q}(U\rho {U}^{\dagger },H\mathrm{).}$$

Axiomatically, the QFI links the fidelity of the distance measurement for two quantum states $\rho (t)$ and $\rho (t+{\rm{\Delta }}t)$ as^26^:

12 $${D}_{B}^{2}(\rho (t),\rho (t+{\rm{\Delta }}t))={F}_{Q}(\rho (t),H){\rm{\Delta }}t+O{({\rm{\Delta }}t)}^{3},$$

with ${D}_{B}^{2}({\rho }_{1},{\rho }_{2})=\mathrm{4(1}-\sqrt{{F}_{B}({\rho }_{1},{\rho }_{2})})$ being the Bures distance, and ${F}_{B}({\rho }_{1},{\rho }_{2})=(Tr\sqrt{\sqrt{{\rho }_{1}}{\rho }_{2}\sqrt{{\rho }_{1}}})$ the Uhlmann fidelity. Here, ${\rho }_{1}=\rho (t)$ and ${\rho }_{2}=\rho (t+{\rm{\Delta }}t)$.

More interestingly, one can always explicitly construct a pure-state ensemble of a given mixed state $\rho ={\sum }_{k}{p}_{k}|{{\rm{\Psi }}}_{k}\rangle \langle {{\rm{\Psi }}}_{k}|$ in the basis $\{|{{\rm{\Psi }}}_{k}\rangle \}$, wherein the QFI in Eq. (9) can be rewritten specifically as^33,34^

13 $${F}_{Q}(\rho ,H)=4\mathop{{\rm{\inf }}}\limits_{\{{p}_{k},|{{\rm{\Psi }}}_{k}\rangle \}}\sum _{k}{p}_{k}{({\rm{\Delta }}H)}_{|{{\rm{\Psi }}}_{k}\rangle }^{2}\mathrm{.}$$

Here, ${({\rm{\Delta }}H)}_{|{{\rm{\Psi }}}_{k}\rangle }^{2}=\langle {{\rm{\Psi }}}_{k}|{H}^{2}|{{\rm{\Psi }}}_{k}\rangle -{\langle {{\rm{\Psi }}}_{k}|H|{{\rm{\Psi }}}_{k}\rangle }^{2}$ is the variance of the observable $H$ for the pure state $|{{\rm{\Psi }}}_{k}\rangle$, and its relevant standard variance reads ${({\rm{\Delta }}H)}_{\rho }^{2}={sup}_{\{{p}_{k},|{\rm{\Psi }}\rangle \}}{p}_{k}{({\rm{\Delta }}H)}_{|{{\rm{\Psi }}}_{k}\rangle }^{2}$. Consequently^33^, we have the following inequality chain:

14 $${F}_{Q}(\rho ,H)\le \sum _{k}{p}_{k}{({\rm{\Delta }}H)}_{|{{\rm{\Psi }}}_{k}\rangle }^{2}\le \mathrm{4(}{\rm{\Delta }}H{)}_{\rho }^{2},$$

where the equality chain holds only for the pure states. Obviously, this inequality chain implies that the upper bound of the QFI is $\mathrm{4(}{\rm{\Delta }}H{)}_{\rho }^{2}$, while the above Cramér-Rao inequality indicates that the lower bound of the QFI is $\mathrm{1/(}{\rm{\Delta }}\theta {)}^{2}$. Given both the lower- and upper bounds of the QFI are observable, quantifying the quantum coherence by the QFI should be physically measurable, at least theoretically.

## Results

We now prove that the QFI satisfies the Baumgratz *et al*.’s criticism and thus can be used to quantify the quantum coherence.

### Verification of condition (C1)

It is easy to prove that the QFI satisfies the condition (C1). In fact, if the density matrix of the state $\rho ={\sum }_{k}{p}_{k}|k\rangle \langle k|$ is diagonal in the eigenvectors $\{|k\rangle \}$ of $H$, then the minimum average of variance of the observable $H$ is ${\sum }_{k}{p}_{k}{({\rm{\Delta }}H)}_{|k\rangle }^{2}=0$, as ${({\rm{\Delta }}H)}_{|k\rangle }^{2}=0$. On the other hand, if the density matrix is not diagonal, then for any decomposition of $\rho$: $\rho ={\sum }_{k}{p}_{k}|{{\rm{\Psi }}}_{k}\rangle \langle {{\rm{\Psi }}}_{k}|$, one can always find a state $|{{\rm{\Psi }}}_{n}\rangle \notin \{|k\rangle \}$ in which ${({\rm{\Delta }}H)}_{|{{\rm{\Psi }}}_{n}\rangle }^{2} > 0$. As a consequence,

15 $$\mathop{{\rm{\inf }}}\limits_{\{{p}_{k},|{{\rm{\Psi }}}_{k}\rangle \}}\sum _{k}{p}_{k}{({\rm{\Delta }}H)}_{|{{\rm{\Psi }}}_{k}\rangle }^{2} > \mathrm{0,}$$

is always satisfied. Therefore, with the Eq. (13) the QFI vanishes if and only if the quantum system is in an incoherent state. This indicates that QFI satisfies the condition (C1) satisfactorily.

### **Verification of condition (C3)**

The convexity of the QFI can be generically expressed as

16 $$\sum {p}_{n}{F}_{Q}({\rho }_{n},H)\ge {F}_{Q}(\sum {p}_{n}{\rho }_{n},H)\triangleq {F}_{Q}^{R}(\rho ,H),$$

with ${p}_{k}\ge 0$, $\sum {p}_{k}\mathrm{=1}$, and ${F}_{Q}^{R}(\tilde{\rho },H)$ being the reduced QFI (to distinguish from the QFI ${F}_{Q}(\rho ,H)$). To verify such a feature, we consider a typical quantum state: $\rho =p{\rho }_{1}+\mathrm{(1}-p){\rho }_{2}$. Obviously, if the decompositions: ${\rho }_{1}={\sum }_{k}{\alpha }_{k}|{\alpha }_{k}\rangle \langle {\alpha }_{k}|$ and ${\rho }_{2}={\sum }_{k}{\beta }_{k}|{\beta }_{k}\rangle \langle {\beta }_{k}|$ satisfy the Eq. (13), then we have ${F}_{Q}({\rho }_{1},H)=4{\sum }_{k}{\alpha }_{k}{({\rm{\Delta }}H)}_{|{\alpha }_{k}\rangle }^{2}$ and ${F}_{Q}({\rho }_{2},H)=4{\sum }_{k}{\beta }_{k}{({\rm{\Delta }}H)}_{|{\beta }_{k}\rangle }^{2}$, respectively. Note that $\rho ={\sum }_{k}p{\alpha }_{k}|{\alpha }_{k}\rangle \langle {\alpha }_{k}|+{\sum }_{k}\mathrm{(1}-p){\beta }_{k}|{\beta }_{k}\rangle \langle {\beta }_{k}|$ is also an effective decomposition of $\rho$, thus

17 $$\begin{array}{rcl}{F}_{Q}^{R}(\rho ,H) & = & {F}_{Q}(p{\rho }_{1}+\mathrm{(1}-p){\rho }_{2},H)\le p{F}_{Q}({\rho }_{1},H)+\mathrm{(1}-p){F}_{Q}({\rho }_{2},H)\\  & = & \sum {p}_{n}{F}_{Q}({\rho }_{n},H\mathrm{).}\end{array}$$

Thus, the QFI is really convex^32^.

### Verification of condition (C2a)

To demonstrate that the QFI satisfies the condition C(2a), i.e., it decreases monotonously under the mixing of density matrix induced by ICPTP operation, we generically introduce a positive linear mapping ${{\mathbb{J}}}_{\rho }^{f}:{{\bf{M}}}_{n}\to {{\bf{M}}}_{n}$. Here, ${{\mathbb{J}}}_{\rho }^{f}=f({{\mathbb{L}}}_{\rho }{{\mathbb{R}}}_{\rho }^{-1}){{\mathbb{R}}}_{\rho }$ with the function $f:{{\mathbb{R}}}^{+}\to {{\mathbb{R}}}^{+}$ being a standard operator monotone function^27^ and ${{\mathbb{L}}}_{\rho }(A)=\rho A$, ${{\mathbb{R}}}_{\rho }(A)=A\rho$. Following the ref.^27^, a $f$-dependent QFI function

18 $${F}_{\rho }^{f}(\theta )=Tr[{\partial }_{\theta }\rho (\theta )({{\mathbb{J}}}_{\rho }^{f}{)}^{-1},({\partial }_{\theta }\rho (\theta \mathrm{))].}$$

is obtained. Here, ${({{\mathbb{J}}}_{\rho }^{f})}^{-1}$ satisfies the following monotonic relation^27,35^: ${{\rm{\Phi }}}^{\ast }{({{\mathbb{J}}}_{{\rm{\Phi }}(\rho )}^{f})}^{-1}{\rm{\Phi }}\le {({{\mathbb{J}}}_{\rho }^{f})}^{-1}$, for any completely positive and trace preserving mapping $\Phi$ defined by Eq. (1). It is proven that^27^

19 $$Tr[{\rm{\Phi }}({\partial }_{\theta }\rho (\theta ))({{\mathbb{J}}}_{{\rm{\Phi }}(\rho )}^{f}{)}^{-1}{\rm{\Phi }}({\partial }_{\theta }\rho (\theta ))]\le Tr[{\partial }_{\theta }\rho (\theta )({{\mathbb{J}}}_{\rho }^{f}{)}^{-1}({\partial }_{\theta }\rho (\theta \mathrm{))].}$$

Thus, QFI is monotonic under the mixing of quantum states^32^. Based on quantum resource theory^6,36^, any function could be used to quantify the quantum coherence, if it satisfies the conditions (C1,C2a) and (C3), simultaneously. A typical example is the distance based on fidelity definition^4^. However, strictly speaking^6^, the function accurately quantifying the quantum coherence should satisfy the condition (C2b), besides the conditions (C1) and (C3). Therefore, the condition (C2b) is stricter than the condition (C2a), and thus is relatively harder to be verified. In the following, we provides such a verification for the QFI under certain assumptions.

### Verification of condition (C2b)

To verify the monotonicity of the QFI, i.e.,

20 $${F}_{Q}(\rho ,H)\ge \sum _{n}{p}_{n}{F}_{Q}({A}_{n}\rho {A}^{\dagger }/{p}_{n},H)\triangleq {F}_{Q}^{A}(\rho ,H),$$

with ${F}_{Q}^{A}(\rho ,H)$ being the average QFI, let us consider a joint quantum system $A+B$. The subsystem $A$ is treated as the work one and the subsystem $B$ the ancillary one, which can be generated by, e.g., the measuring apparatus or the environment of the subsystem $A$. Suppose that $A+B$ is closed and thus any dynamic process of such a joint quantum system can be described by a unitary evolution, i.e., ${\rho }_{AB}(t)=U{\rho }_{AB}\mathrm{(0)}{U}^{\dagger }$. By taking partial trace on the subsystem $B$, then the reduced density matrix of the subsystem $A$ at time $t$ is given as ${\rho }_{A}(t)=T{r}_{B}[U{\rho }_{AB}\mathrm{(0)}{U}^{\dagger }]$. Typically, for the initial state of the joint system ${\rho }_{AB}\mathrm{(0)}={\rho }_{A}\mathrm{(0)}\otimes {\rho }_{B}\mathrm{(0)}$, the state of the subsystem $A$ at the time $t > 0$ takes consequently the form in Eq. (1), with ${A}_{\mu }^{\dagger }={A}_{ij}^{\dagger }=\sqrt{{\lambda }_{i}}\langle {\psi }_{i}|U|{\psi }_{j}\rangle$. Here, $\{{\lambda }_{i},|{\psi }_{i}\rangle \}$ is a spectral decomposition of ${\rho }_{B}\mathrm{(0)}$, i.e., ${\rho }_{B}\mathrm{(0)}={\sum }_{i}{\lambda }_{i}|{\psi }_{i}\rangle \langle {\psi }_{i}|$. On the other hand, it has been proven that^24,25^, one can always construct an extended state $\rho \mathrm{(0)}={\rho }_{A}\otimes |\psi {\rangle }_{B}\langle \psi |$ and a unitary transformation $U$ to satisfy the dynamical map Eq. (1). Here, $|\psi {\rangle }_{B}$ is fixed for any state ${\rho }_{A}$ of the subsystem $A$, and ${A}_{\mu }{=}_{B}{\langle {\varphi }_{\mu }|U|\psi \rangle }_{B}$ with ${\{|{\varphi }_{\mu }\rangle }_{B}\}$ being the basis of the Hilbert space for the subsystem $B$.

First, if the system is initially in a pure state $|{\psi }_{i}\rangle =|\psi {\rangle }_{A}\otimes |\psi {\rangle }_{B}$, then under the unitary operation $U$, it will evolve generically to $|{\psi }_{f}\rangle ={\sum }_{kl}\varphi (k,l)|{\alpha }_{k}\rangle |{\beta }_{l}\rangle$ with $|{\alpha }_{k}\rangle$ and $|{\beta }_{l}\rangle$ being the orthogonal eigenvectors of Hamiltonian ${H}_{A}$ and ${H}_{B}$, respectively. This means that, the QFI in the pure state $|\psi {\rangle }_{f}$ can be easily calculated as

21 $${F}_{Q}(|{\psi }_{f}\rangle ,{H}_{A}\otimes {I}_{B}+{I}_{A}\otimes {H}_{B})=4\{\sum _{kl}p(k,l){{\rm{\Gamma }}}_{kl}^{2}-{[\sum _{kl}p(k,l){{\rm{\Gamma }}}_{kl}]}^{2}\},$$

where $p(k,l)=|\varphi (k,l{)|}^{2}$ and ${{\rm{\Gamma }}}_{kl}={\alpha }_{k}+{\beta }_{l}$. Suppose that ${H}_{A}\otimes {I}_{B}+{I}_{A}\otimes {H}_{B}$ commutes with the unitary transformation $U$, then from Eqs (10) and (11), we have ${F}_{Q}(|{\psi }_{f}\rangle ,{H}_{A}\otimes {I}_{B}+{I}_{A}\otimes {H}_{B})={F}_{Q}{(|\psi \rangle }_{A},{H}_{A})+{F}_{Q}{(|\psi \rangle }_{B},{H}_{B})$, and ${F}_{Q}{(|\psi \rangle }_{A},{H}_{A})=\mathrm{4\{}{\sum }_{kl}p(k,l){\alpha }_{k}^{2}-{[{\sum }_{kl}p(k,l){\alpha }_{k}]}^{2}\}$. This implies that, if one performs a measurement on the subsystem $B$ and obtain the outcome ${\beta }_{l}$, then the joint quantum system will collapse into the state

22 $$|{\psi }_{f}(l)\rangle =\frac{1}{\sqrt{p(l)}}\sum _{k}\varphi (k,l)|{{\alpha }}_{k}\rangle \otimes |{{\beta }}_{l}\rangle $$

with $p(l)={\sum }_{k}|\varphi (k,l{)|}^{2}$. As the probability to find the subsystem $B$ in state $|{\beta }_{l}\rangle$ is $p(l)$, the average QFI of the joint system $A+B$ after the subselection, related to the measurement outcome, can be calculated as ${F}_{Q}(H)={\sum }_{l}p(l){F}_{Q}(|{\psi }_{f}(l)\rangle ,H)$ with ${F}_{Q}(|{\psi }_{f}(l)\rangle ,H)=\mathrm{4(}\Delta H{)}_{|{\psi }_{f}(l)\rangle }^{2}$ being the QFI of the state $|{\psi }_{f}(l)\rangle$. Furthermore, with the help of Eqs (21) and (22), we have ${F}_{Q}(H)=\mathrm{4[}{\sum }_{kl}p(k,l){\alpha }_{k}^{2}-{\sum }_{l}{({\sum }_{k}p(k,l){\alpha }_{k})}^{2}]/p(l)$. After a straightforward derivation, one can verify that^37^

23 $${F}_{Q}{(|\psi \rangle }_{A},{H}_{A})-{F}_{Q}(H)=\sum _{l}{\{\sum _{k}[\frac{p(k,l)}{\sqrt{p(l)}}-\sqrt{p(l)}\mathrm{.}\sum _{{l}^{\text{'}}}p(k,{l}^{\text{'}})]{\alpha }_{k}\}}^{2}\ge 0$$

This indicates that the QFI is really nonincreasing, as ${F}_{Q}(H)$ is actually just the statistical average of ${F}_{Q}({\rho }_{l},{H}_{A})$, i.e., ${F}_{Q}({\rho }_{l},{H}_{A})={\sum }_{l}{p}_{l}{F}_{Q}({\rho }_{l},{H}_{A})$ with ${\rho }_{l}={A}_{l}|\psi {\rangle }_{A}\langle \psi |{A}_{l}^{\dagger }/{p}_{l}$ and ${A}_{l}={}_{B}{\langle {\beta }_{l}|U|\psi \rangle }_{B}$. Therefore, the monotonicity of the QFI

24 $${F}_{Q}{(|\psi \rangle }_{A},{H}_{A})\ge \sum _{l}{p}_{l}{F}_{Q}({A}_{l}|\psi \rangle \langle \psi |{A}_{l}^{\dagger }/{p}_{l},{H}_{A})\triangleq {F}_{Q}^{A}{(|\psi \rangle }_{A},{H}_{A}),$$

is verified.

Next, for a more generic initial state, e.g., ${\rho }_{A}\times |\psi {\rangle }_{BB}\langle \psi |$ with the subsystem $A$ being in a mixture one: ${\rho }_{A}={\sum }_{k}{w}_{k}|{w}_{k}\rangle \langle {w}_{k}|$, we have ${F}_{Q}({\rho }_{A},{H}_{A})={\sum }_{k}{w}_{k}{F}_{Q}(|{w}_{k}\rangle ,{H}_{A})$, as the decomposition $\{{w}_{k},|{w}_{k}\rangle \}$ fulfills the Eq. (13). Furthermore, with Eq. (24), we have

25 $${F}_{Q}({\rho }_{A},{H}_{A})\ge \sum _{k}{w}_{k}\sum _{l}{p}_{l}{F}_{Q}({A}_{l}|{w}_{k}\rangle \langle {w}_{k}|{A}_{l}^{\dagger }/{p}_{l},{H}_{A}\mathrm{).}$$

Finally, from the convexity of the QFI verified above, one can prove that

26 $${F}_{Q}({\rho }_{A},H)\ge \sum _{l}{p}_{l}{F}_{Q}({A}_{l}{\rho }_{A}{A}_{l}^{\dagger }/{p}_{l},H)\triangleq {F}_{Q}^{A}({\rho }_{A},H\mathrm{).}$$

This indicates that the QFI satisfies the condition (C2b) specifically.

## Numerical confirmations

The validity of the above verifications can be more clearly demonstrated with certain specific incoherent processes. Without loss of the generality, let us consider the QFI in a generic one-qubit state

27 $$\tilde{\rho }=\frac{1}{2}(I+a{\sigma }_{x}+b{\sigma }_{y}+c{\sigma }_{z})\triangleq \frac{1}{2}(I+\overrightarrow{n}\cdot \overrightarrow{\sigma }),$$

with $\overrightarrow{n}=(a,b,c)$ and $\overrightarrow{\sigma }=({\sigma }_{x},{\sigma }_{y},{\sigma }_{z}{)}^{\dagger }$. Obviously, the eigenvalues of such a density operator can be easily obtained as ${\lambda }_{1}=\mathrm{(1}+|n\mathrm{|)/2,}\,\,|n|=\sqrt{{a}^{2}+{b}^{2}+{c}^{2}}$ and ${\lambda }_{2}=\mathrm{(1}-|n\mathrm{|)/2}$, with the corresponding eigenvectors being $|{\lambda }_{1}\rangle =(a+ib,|n|-c{)}^{\dagger }/\sqrt{\mathrm{2|}n{|}^{2}-2c}$ and $|{\lambda }_{2}\rangle =(a+ib,-|n|-c{)}^{\dagger }/\sqrt{\mathrm{2|}n{|}^{2}+2c}$, respectively.

### **The** convexity and monotonicity of the QFI, i.e., the Eqs (16) and (20)

First, let us consider a depolarizing process, which can be described equivalently by the following incoherent operation (with $p\in \mathrm{[0,}\,\mathrm{1]}$):

28 $${A}_{0}=\sqrt{1-p}I\mathrm{,\ }{A}_{1}=\sqrt{\frac{p}{3}}{\sigma }_{x}\mathrm{,\ }{A}_{2}=\sqrt{\frac{p}{3}}{\sigma }_{y}\mathrm{,\ }{A}_{3}=\sqrt{\frac{p}{3}}{\sigma }_{z},$$

in the Kraus representation. One can easily prove that, for the mixture state (27) an ICPTP could be constructed by the following unitary transformation:

29 $$\begin{array}{rcl}{U}_{d} & = & \sqrt{1-p}{I}^{A}\otimes ({\sigma }_{00}^{B}-{\sigma }_{22}^{B})+\sqrt{1-p}({\sigma }_{00}^{A}-{\sigma }_{11}^{A})\otimes ({\sigma }_{11}^{B}-{\sigma }_{33}^{B})\\  &  & +\frac{p}{3}[{\sigma }_{00}^{A}\otimes ({\sigma }_{03}^{B}+{\sigma }_{30}^{B}+{\sigma }_{12}^{B}+{\sigma }_{21}^{B})+{\sigma }_{11}^{A}\otimes ({\sigma }_{03}^{B}-{\sigma }_{30}^{B}-{\sigma }_{12}^{B}+{\sigma }_{21}^{B})\\  &  & +{\sigma }_{01}^{A}\otimes ({\sigma }_{01}^{B}+{\sigma }_{10}^{B}-{\sigma }_{02}^{B}+{\sigma }_{20}^{B}-{\sigma }_{13}^{B}+{\sigma }_{31}^{B}+{\sigma }_{23}^{B}+{\sigma }_{32}^{B})\\  &  & +{\sigma }_{10}^{A}\otimes ({\sigma }_{01}^{B}\,+\,{\sigma }_{10}^{B}-{\sigma }_{02}^{B}-{\sigma }_{20}^{B}-{\sigma }_{13}^{B}-{\sigma }_{31}^{B}-{\sigma }_{23}^{B}-{\sigma }_{32}^{B})],\end{array}$$

with $|\psi {\rangle }_{B}=\mathrm{|0}{\rangle }_{B}$, $|{\varphi }_{2}{\rangle }_{B}=i\mathrm{|2}{\rangle }_{B}$, ${\sigma }_{ij}^{A,B}=|i{\rangle }_{A,B}\langle j|$, and $|{\varphi }_{\mu }{\rangle }_{B}=|\mu {\rangle }_{B},\mu =\mathrm{0,}\,\mathrm{1,}\,3$. Here, $|i{\rangle }_{A}$ and $|i{\rangle }_{B}$ are the basis of the Hilbert spaces for the subsystems A and B, respectively. Certainly, the above unitary transformation ${U}_{d}$, satisfying the relation ${A}_{\mu }\,={}_{B}{\langle {\varphi }_{\mu }|{U}_{d}|\psi \rangle }_{B}$, is not unique. By utilizing Eq. (9) one can easily check that

30 $${F}_{Q}(\tilde{\rho },H)={a}^{2}+{b}^{2},$$

with $H=\mathrm{|0}\rangle \langle \mathrm{0|}$. It is easy to prove that, ${\tilde{\rho }}_{\mu }={A}_{\mu }\tilde{\rho }{A}_{\mu }^{\dagger }/{p}_{\mu },\,\mu =\mathrm{0,}\,\mathrm{1,}\,\mathrm{2,}\,3$ with ${p}_{\mu }=Tr({A}_{\mu }\tilde{\rho }{A}_{\mu }^{\dagger })$ and ${\sum }_{\mu }{A}_{\mu }\tilde{\rho }{A}_{\mu }^{\dagger }=$ $(I+\mathrm{(1}-4p\mathrm{/3)}n\cdot \overrightarrow{\sigma })$. Thus, the average QFI in Eq. (26) and the reduced QFI in Eq. (16) for the present state (27) are easily calculated as

31 $${F}_{Q}^{A}(\tilde{\rho },H)={a}^{2}+{b}^{2},$$

and

32 $${F}_{Q}^{R}(\tilde{\rho },H\mathrm{)=(1}-4p{\mathrm{/3)}}^{2}({a}^{2}+{b}^{2}),$$

respectively.

Similarly, for the phase damping channel with the equivalent ICPTP

33 $${A}_{0}=\sqrt{1\,-\,p}I\mathrm{,\ }{A}_{1}=\sqrt{p}[\begin{array}{cc}1 & 0\\ 0 & 0\end{array}]\mathrm{,\ }{A}_{2}=\sqrt{p}[\begin{array}{cc}0 & 0\\ 0 & 1\end{array}],$$

a unitary transformation:

34 $$\begin{array}{rcl}{U}_{p} & = & \mathrm{|0}{\rangle }_{A}\langle \mathrm{0|}\otimes (\sqrt{1-p}\mathrm{|0}{\rangle }_{B}{\langle \mathrm{0|}-\sqrt{1-p}\mathrm{|1}\rangle }_{B}{\langle \mathrm{1|}+\mathrm{|2}\rangle }_{B}\langle \mathrm{2|})\\  &  & +\mathrm{|1}{\rangle }_{A}\langle \mathrm{1|}\otimes (\sqrt{1-p}\mathrm{|0}{\rangle }_{B}{\langle \mathrm{0|}+\mathrm{|1}\rangle }_{B}{\langle \mathrm{1|}-\sqrt{1-p}\mathrm{|2}\rangle }_{B}\langle \mathrm{2|})\\  &  & +\sqrt{p}(\mathrm{|0}{\rangle }_{A}{\langle \mathrm{0|}\otimes \mathrm{|0}\rangle }_{B}{\langle \mathrm{1|}+\mathrm{|1}\rangle }_{A}{\langle \mathrm{1|}\otimes \mathrm{|0}\rangle }_{B}\langle \mathrm{2|}+C\mathrm{.}C),\end{array}$$

with $|\psi {\rangle }_{B}=|0{\rangle }_{B}$, $|{\phi }_{\mu }{\rangle }_{B}=|\mu {\rangle }_{B}$, can be constructed. Correspondingly, we have ${\sum }_{\mu }{A}_{\mu }\tilde{\rho }{A}_{\mu }^{\dagger }=(I+\mathrm{(1}-p)$ $a{\sigma }_{x}+\mathrm{(1}-p)b{\sigma }_{y}+c{\sigma }_{z}\mathrm{)/2}$. As a consequence, the average QFI in Eq. (26) and the reduced QFI in Eq. (16) read

35 $${F}_{Q}^{A}(\tilde{\rho },H)=\mathrm{(1}-p){F}_{Q}(\tilde{\rho },H)\le {F}_{Q}(\tilde{\rho },H),$$

and

36 $${F}_{Q}^{R}(\tilde{\rho },H)={\mathrm{(1}-p)}^{2}{F}_{Q}(\rho ,H),$$

respectively.

**Figure 1:**
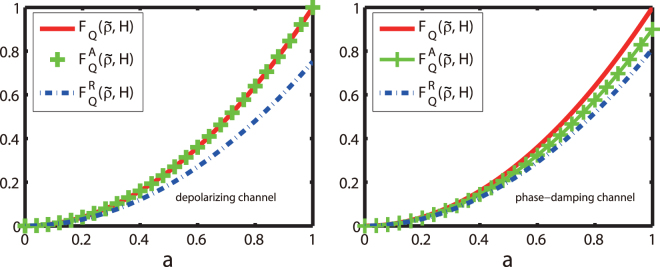
(Color online) The monotonicity and convexity of the QFI for the typical depolarizing- and phase-damping processes (versus the parameter $a$ in the generic one-qubit state with $b=0$, $c=\sqrt{1-{a}^{2}}$ and $p=0.1$): the QFI function ${F}_{Q}(\tilde{\rho },H)$ (red solid), the average QFI function ${F}_{Q}^{A}(\tilde{\rho },H)$ (green plus sign) in Eq. (26) and the reduced QFI function ${F}_{Q}^{R}(\tilde{\rho },H)$ (blue dashed-dotted) in Eq. (16).

Figure 1 shows how the QFI, the average QFI, and the reduced QFI functions vary with the parameter $a$ in the quantum state $\tilde{\rho }$. It is seen that, for both the depolarizing- and the phase-damping processes described here, these functions are all monotonic and convex. Specifically, for any parameter $a$, Eqs (16) and (20) are always established. This clearly indicates that the QFI satisfies the Baumgratz *et al*.’s criticism and thus can be utilized to quantify the quantum coherence, at least theoretically.

### Comparisons with the other measure methods

To further check the validity of our proposal, we compare the QFI with the other functions proposed previously for quantifying quantum coherence. Specifically, for a common single-qubit quantum state (27) with ${a}^{2}+{b}^{2}+{c}^{2}\le 1$, the relative entropy^6^ are calculated as

37 $$\begin{array}{rcl}{C}_{r}(\tilde{\rho }) & = & \mathop{{\rm{\min }}}\limits_{\hat{\delta }\in {\rm{II}}}\,S(\rho \parallel \hat{\delta })\\  & = & \frac{1}{2}[\mathrm{(1}+n)\,{\mathrm{log}}_{2}\,\mathrm{(1}+n)+\mathrm{(1}-n)\,{\mathrm{log}}_{2}\,\mathrm{(1}-n)\\  &  & -\mathrm{(1}+c)\,{\mathrm{log}}_{2}\,\mathrm{(1}+c)-\mathrm{(1}-c)\,{\mathrm{log}}_{2}\,\mathrm{(1}-c)].\end{array}$$

Analogously, the fidelity based on distance measurement defined by^4^ ${C}_{f}(\tilde{\rho })=1-\sqrt{{{\rm{\max }}}_{\delta \in I}F(\tilde{\rho },\delta )}$ with $F(\tilde{\rho },\delta )=[tr\sqrt{{\tilde{\rho }}^{\mathrm{1/2}}\delta {\tilde{\rho }}^{\mathrm{1/2}}}]$ can be expressed as

38 $${C}_{f}(\tilde{\rho })=1-\frac{\sqrt{2}}{2}\sqrt{1+\sqrt{1-{a}^{2}-{b}^{2}}},$$

and the ${l}_{1}\,norms$ function reads

39 $${C}_{{l}_{1}}(\tilde{\rho })=\sum _{i,j(i\ne j)}|{\tilde{\rho }}_{ij}|=\sqrt{{a}^{2}+{b}^{2}}\mathrm{.}$$

**Figure 2:**
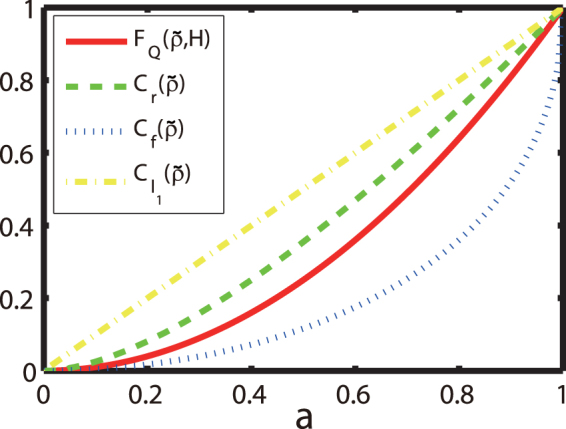
(Color online) Quantum coherence in a typical one-qubit state (27) quantified by different functions; the QFI (red solid), relative entropy (green dashed), fidelity (blue dotted) and ${l}_{1}\,norm$ ${C}_{{l}_{1}}$ (yellow dashed-dotted). Here, for simplicity the parameter $a$ in (27) is changed from $0$ to $1$, $b$ is fixed as $0$, and $c=\sqrt{1-{a}^{2}}$. For comparison, all the values of these functions are normalized by divided their achievable maximum.

It is seen from the Fig. 2 that, all of these functions really measure the quantum coherence; different coherent suppositions (with different parameters $a$) correspond to different values of the quantifying functions. When $a$ equals $0$ (which corresponds to a completely-mixed state), the values of these functions equal to $0$; While, for the typical supposition pure state $\mathrm{(|0}\rangle +\mathrm{|1}\rangle )/\sqrt{2}$, they all reach a common normalized maximum value $1$.

## Conclusion

In summary, we have verified that the QFI could satisfy the Baumgratz *et al*.’s criticism and thus can also be utilized to quantify the quantum coherence. Given most of the other coherence measurements proposed previously, e.g., the relative entropy, fidelity, and ${l}_{1}\,norms$ etc., are basically axiomatic, the QFI quantification of the quantum coherence seems more experimental, as its lower- and upper bounds are both related to certain measurable quantities.
